# The complete mitochondrial genome of the silver-backed chevrotain (*Tragulus versicolor*) from Vietnam and its phylogenetic position within Tragulidae

**DOI:** 10.1080/23802359.2025.2572391

**Published:** 2025-10-13

**Authors:** Huy Hoang Quoc, Long Ha Thang, Mau Trinh Dang, Son Nguyen Truong, Dac Chung Ngo

**Affiliations:** ^a^ GreenViet Biodiversity Conservation Centre, Da Nang City, Vietnam; ^b^ University of Education, Hue University, Hue City, Vietnam; ^c^ University of Science and Education, Da Nang University, Da Nang City, Vietnam; ^d^ Institute of Biology (VAST), Hanoi City, Vietnam

**Keywords:** Mitogenome, mouse deer, phylogeny, endemic ungulate, Southeast Asia

## Abstract

The silver-backed chevrotain (*Tragulus versicolor* Thomas, 1910) is the only endemic ungulate species in Vietnam and among the most little-known ungulates in the world. The species currently has a very narrow distribution area and is facing many threats of extinction due to habitat loss and illegal hunting. In this study, we describe complete mitochondrial genome of *T. versicolor* and its taxonomic position within Tragulidae. The circular genome is 16,317 bp in length, comprising 13 protein-coding genes, 22 tRNA genes, two rRNA genes, and one control region, with a GC content of 40.3%. Phylogenetic analysis based on complete mitochondrial genomes using the maximum-likelihood method confirms the monophyly of Tragulidae and the distinct position of *T. versicolor* within *Tragulus*. This is the first publicly available mitogenome of *T. versicolor* (GenBank accession no. PV872897), which provides an essential resource for future studies on evolution, taxonomy, and conservation of this elusive species.

## Introduction

**Figure 1.**
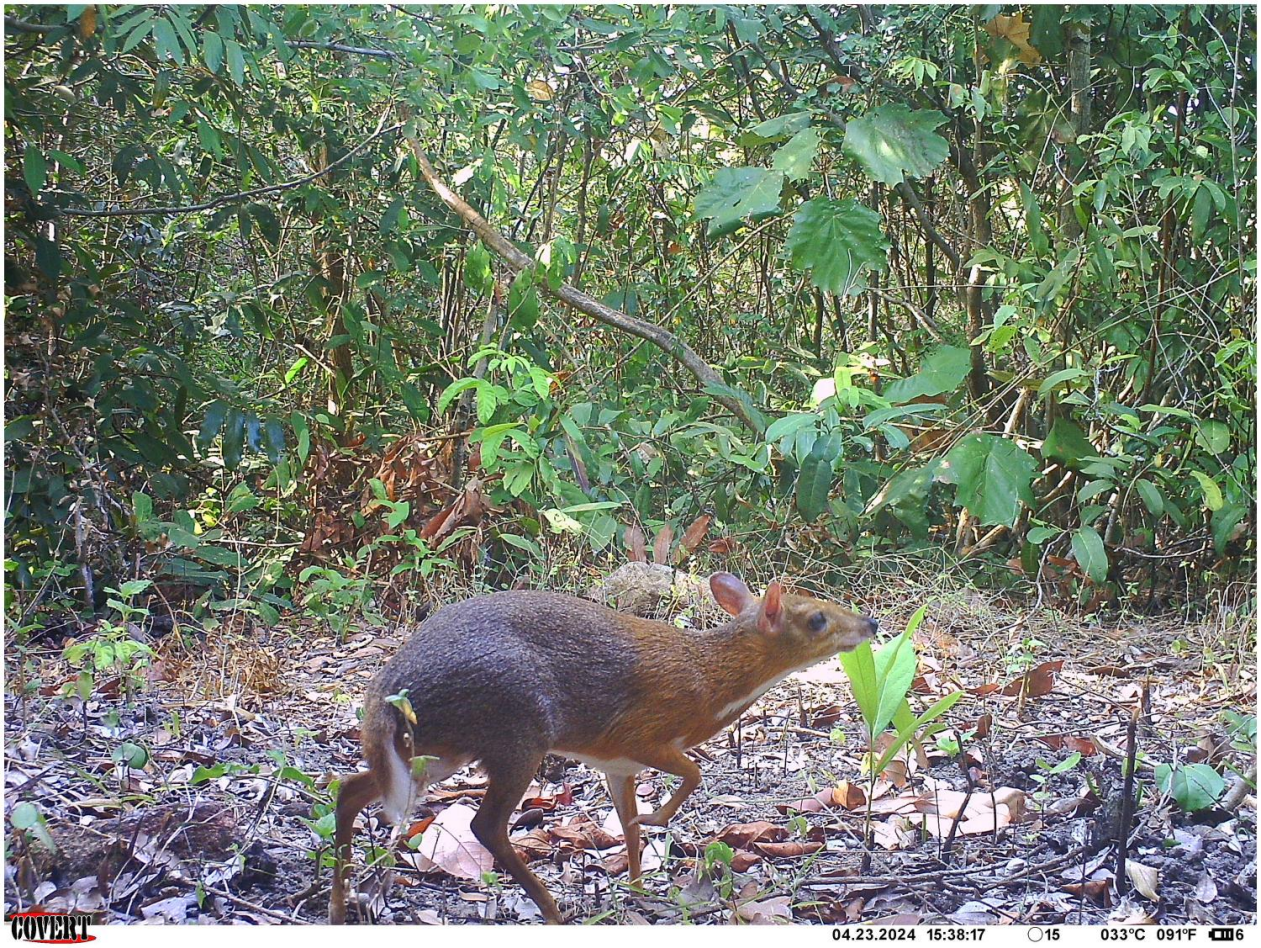
Camera trap photo of *Tragulus versicolor* taken in Khanh Hoa Province, Vietnam (2024). Photo credit: Leibniz-IZW/SIE/GreenViet.

The silver-backed chevrotain (*Tragulus versicolor* Thomas, 1910) (Figure 1), also known as the Vietnam chevrotain, is a critically endangered species according to the Vietnam Red Book (Dang et al. 2023). The species was first described by Thomas in 1910 based on specimens collected near Nha Trang City, southern Vietnam (Thomas 1910). After a long period with no confirmed records, a single confirmed specimen of *T. versicolor* was recorded in Gia Lai Province in 1990, in sympatry with *T. kanchil* (Kuznetsov and Borissenko 2004). *T. versicolor* then remained undetected until 2017, when the first photographs of a wild population were obtained using camera traps in Nui Chua National Park (Nguyen et al. 2019). *T. versicolor* is characterized by a distinctive two-tone pelage – rufous anterior and silvery posterior – that distinguishes it from other members of the genus *Tragulus* (Thomas 1910).

The silver-backed chevrotain has a highly restricted distribution in dry and semi-dry forests of south-central Vietnam, where recent field surveys confirmed five populations across a narrow coastal range (Dang et al. 2023). Here, we present the first complete mitochondrial genome of *T. versicolor*, providing a valuable reference for phylogenetic analysis and a baseline resource for conservation genetics of this evolutionarily distinct and threatened species.

## Materials and methods

### Sample collection

A tissue sample of *T. versicolor* was collected on 24 October 2022 from Ninh Hoa District, Khanh Hoa Province, Vietnam (12.4911°N, 109.1906°E). The specimen was obtained from a local hunter under the permission and supervision of local rangers. The species was identified based on external morphology, including the two-tone pelage pattern – rufous anterior and silvery posterior – and the size consistent with a mature individual (Thomas 1910). A muscle tissue sample was preserved in 95% ethanol and stored at −20 °C until DNA extraction. The specimen was deposited at the Department of Zoology, University of Science, Vietnam National University, Hanoi (https://en.vnu.edu.vn/university-of-science-vnuhus-post12712.html; contact: Dr. Nguyen Thi Tham, thamnguyenclthp@gmail.com) under the voucher number KH01.

### DNA extraction and sequencing

Genomic DNA was extracted using the QIAamp DNA Mini Kit (Qiagen, Hilden, Germany). A paired-end library (PE150) was constructed following the DNBSEQ™ protocol and sequenced on the DNBSEQ platform at BGI Genomics (Shenzhen, China), yielding 4,042,092 clean reads (∼1.21 Gb). The clean reads exhibited high quality, with 98.77% of bases having a Phred score ≥ Q20, and 95.73% ≥ Q30. Adapters and low-quality reads were removed using SOAPnuke v1.5.6 with the following parameters: -n 0.01 -l 20 -q 0.5 -adaMR 0.25 --polyX 50 --minReadLen 150 (Chen et al. 2018). Clean data were used for subsequent mitochondrial genome assembly. Clean data were checked with FastQC v0.11.9 to review base composition and score profiles.

To verify congruence between reads and the circular assembly, the clean reads were remapped to a circularized reference with the origin placed within the control region using BWA-MEM v0.7.17 (Li 2013); alignments were processed with samtools v1.19 (Danecek et al. 2021). Per-base depth was computed with samtools depth -a -J and used to generate the coverage profile and histogram (Supplementary Figure S1). This analysis indicated a mean coverage ∼254× across the mitogenome and a breadth (≥1×) of 99.8%.

### Genome assembly and annotation

The mitochondrial genome was assembled de novo using GetOrganelle v1.7.7 (Jin et al. 2020) with the animal_mt setting and *T. kanchil* as a reference. Assembly quality was verified by checking for internal stop codons, and gene positions. Annotation was performed using the MITOS2 web server (Donath et al. 2019) with the vertebrate mitochondrial code (transl_table = 2). In total, 37 genes were identified, including 13 protein-coding genes (PCGs), 22 tRNAs, and two rRNAs. Gene boundaries, orientations, and codons were manually curated in Geneious Prime v2025.1.3 (Biomatters Ltd., Auckland, New Zealand), and the final circular mitogenome map was generated in Geneious.

### Phylogenetic analysis

Complete mitochondrial genomes of five other Tragulidae were retrieved from GenBank: *Tragulus kanchil* (JN632709), *T. napu* (KY117549), *T. javanicus* (LN873134), *Hyemoschus aquaticus* (JN632650), *Moschiola indica* (KY290452), and *Bos taurus* (AY526085) as the outgroup. Complete mitochondrial genomes, including all 37 genes (13 PCGs, 22 tRNAs, and two rRNAs) and the control region (D-loop), were aligned as a whole using MAFFT v7.490 (Katoh and Standley 2013) with the L-INS-i algorithm. A maximum-likelihood tree was reconstructed in IQ-TREE v2.2.2.6 (Minh et al. 2020) under the GTR + F + I + G4 model with 1000 ultrafast bootstrap replicates. The tree was rooted with *Bos taurus* and visualized in iTOL v7.2.1 (Letunic and Bork 2021).

## Results

**Figure 2.**
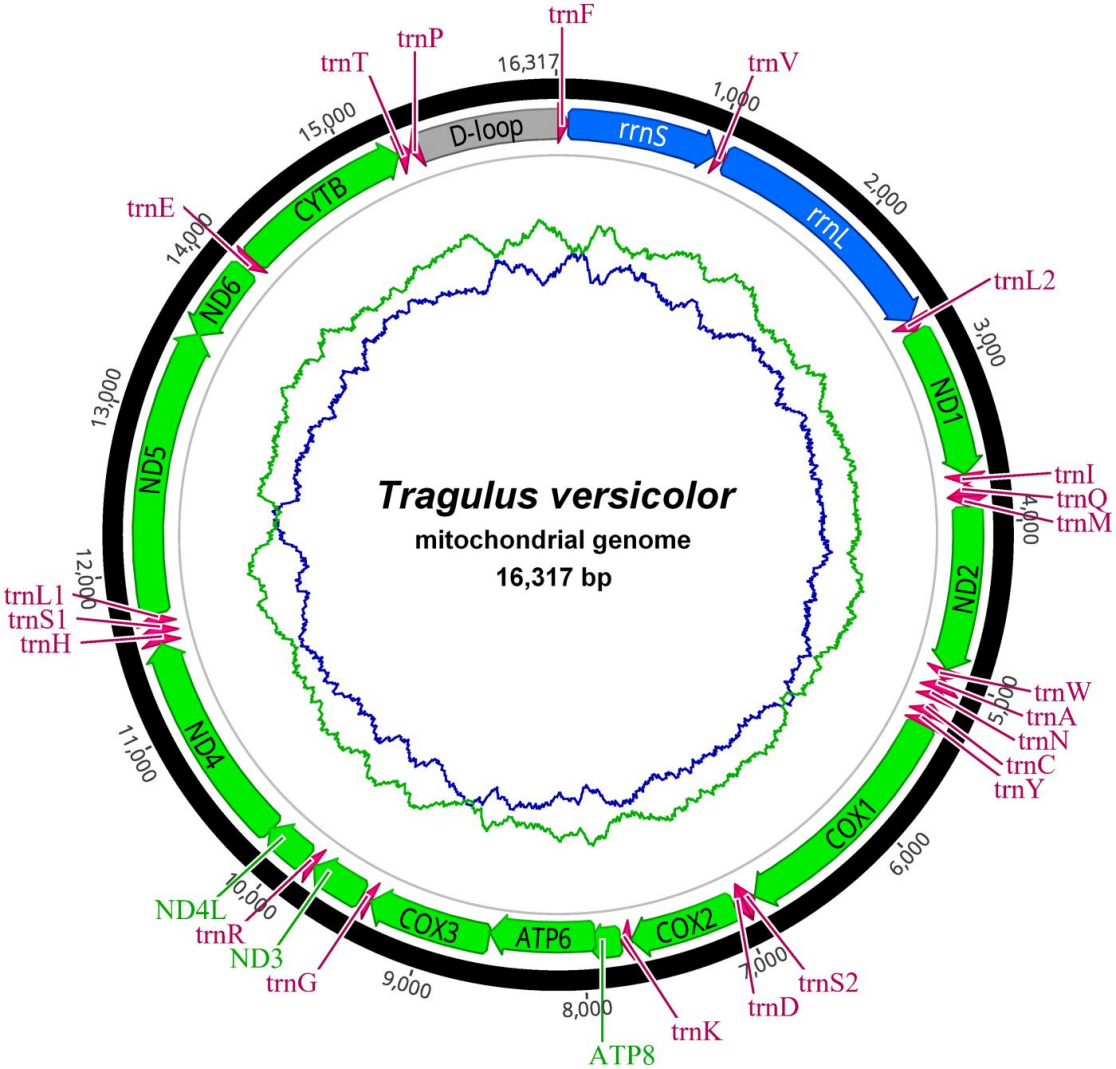
Circular mitochondrial genome map of *Tragulus versicolor*. Protein-coding genes in green, tRNAs in pink, rRNAs in blue, and the control region (D-loop) in gray. The GC-skew in green and AT-skew in blue curves.

The complete mitochondrial genome of *T. versicolor* comprises 37 genes, including 13 PCGs, 22 tRNA genes, and two rRNA genes (Figure 2). Among these, the majority are encoded on the heavy (H) strand, while eight tRNA genes and *nad6* are located on the light (L) strand. Detailed gene coordinates (start–end), strand orientation, and tRNA anticodons are available in the GenBank record for this mitogenome (accession PV872897). The overall base composition is 32.7% A, 26.9% T, 26.8% C, and 13.6% G. The GC content is 40.3%.

**Figure 3.**
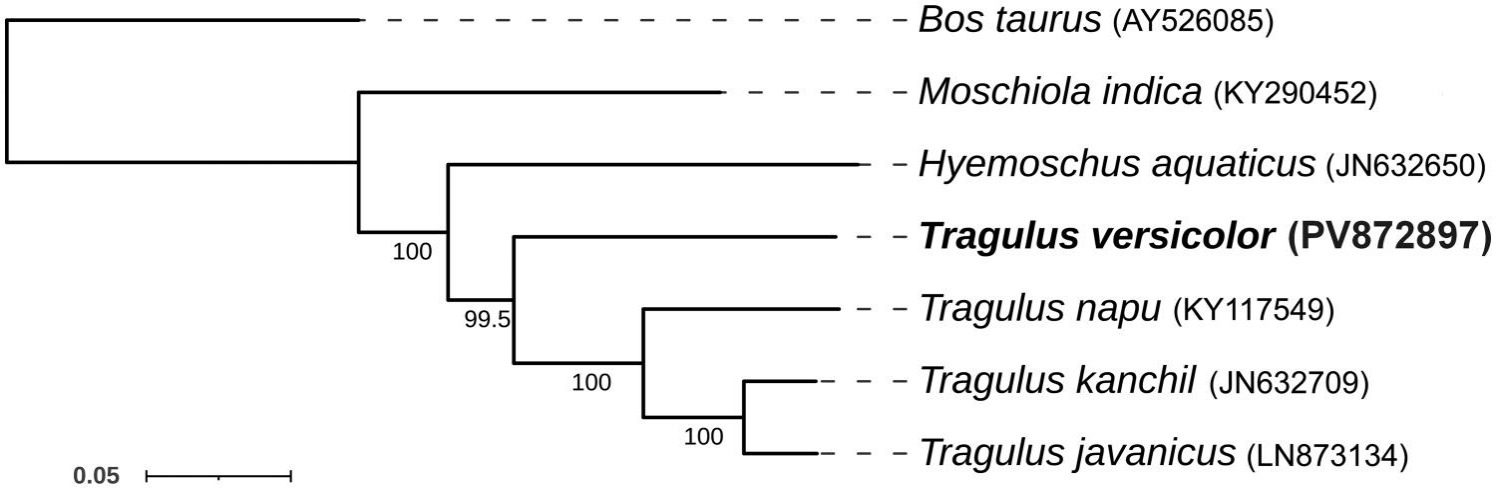
Phylogram based on the maximum-likelihood analysis of complete mitochondrial genomes. Numbers at nodes represent bootstrap values (>50%). the following sequences were used: AY526085 (Chung and Ha 2004), KY290452 (Sarvani et al. 2018), JN632650 (Hassanin et al. 2012), KY117549 (Mohd Salleh et al. 2017), JN632709 (Hassanin et al. 2012), and LN873134 (Gallus et al. 2015).

The phylogenetic analysis of complete mitochondrial genomes by maximum-likelihood produced a well-resolved topology with strong bootstrap support (Figure 3). *Tragulus versicolor* formed a distinct lineage, sister to the clade comprising *T. napu*, *T. kanchil*, and *T. javanicus*, with a bootstrap value of 100%. This indicates an early divergence of *T. versicolor* from the remaining Southeast Asian tragulids.

## Discussion and conclusions

Gene sizes in *T. versicolor* are consistent with other tragulid mitogenomes, with cox1 being the longest PCG (1545 bp) and atp8 the shortest (207 bp), a pattern also observed in *T. kanchil* (Abu-Bakar et al. 2024). The rRNA genes (*rrnS* and rrnL) lie between *trnF* and *trnL2*, and the control region (D-loop) between *trnP* and *trnF*, conforming to the canonical vertebrate order (Hassanin et al. 2012; Sarvani et al. 2018). This arrangement matches those of *T. kanchil*, *T. napu*, and *T. javanicus* (Endo et al. 2004; Mohd Salleh et al. 2017; Abu-Bakar et al. 2024), indicating a conserved mitochondrial genome structure across *Tragulus*.

Phylogenetic analysis confirmed the basal position of *Moschiola indica*, in line with earlier cytb and mitogenome studies (Hassanin et al. 2012; Sarvani et al. 2018; Abu-Bakar et al. 2024). The outgroup *Bos taurus* was clearly separated, supporting the monophyly of Tragulidae and the placement of *T. versicolor* within it. Compared to Bayesian inference using cytb (Nguyen et al. 2024), our ML mitogenome analysis provided stronger support, showing *T. versicolor* as a distinct lineage with an intermediate branch length.

The complete mitogenome of the silver-backed chevrotain provides an essential genetic resource for species identification, forensic analysis, and conservation planning. As an evolutionarily distinct lineage needing urgent protection, these data will aid law enforcement against illegal trade and guide future assessments of population structure and genetic diversity for conservation management.

## Supplementary Material

Supplementary Figure S1.pdf

## Data Availability

The genome sequence data that support the findings of this study are openly available in GenBank of NCBI at https://www.ncbi.nlm.nih.gov under the accession no. PV872897. The associated BioProject, SRA, and BioSample numbers are PRJNA1303987, SRR34936334, and SAMN50545290, respectively.
