# Supplementary material for: The complete mitochondrial genome of the silver-backed chevrotain (Tragulus versicolor) from Vietnam and its phylogenetic position within Tragulidae
Source: Mitochondrial DNA B Resour. 2025 Oct 13;10(11):1042–5. doi: 10.1080/23802359.2025.2572391 (PMC12570224; doi:10.1080/23802359.2025.2572391)
Supplement: Supplementary Figure S1.pdf [file TMDN_A_2572391_SM0187.pdf]

Supplementary Material

This supplementary file belongs to the manuscript: “The complete mitochondrial genome of the Silver-backed chevrotain (*Tragulus versicolor*) from Vietnam and its phylogenetic position within Tragulidae”.

Authors: Huy Hoang Quoc, Long Ha Thang, Mau Trinh Dang, Son Nguyen Truong, Chung Ngo Dac.

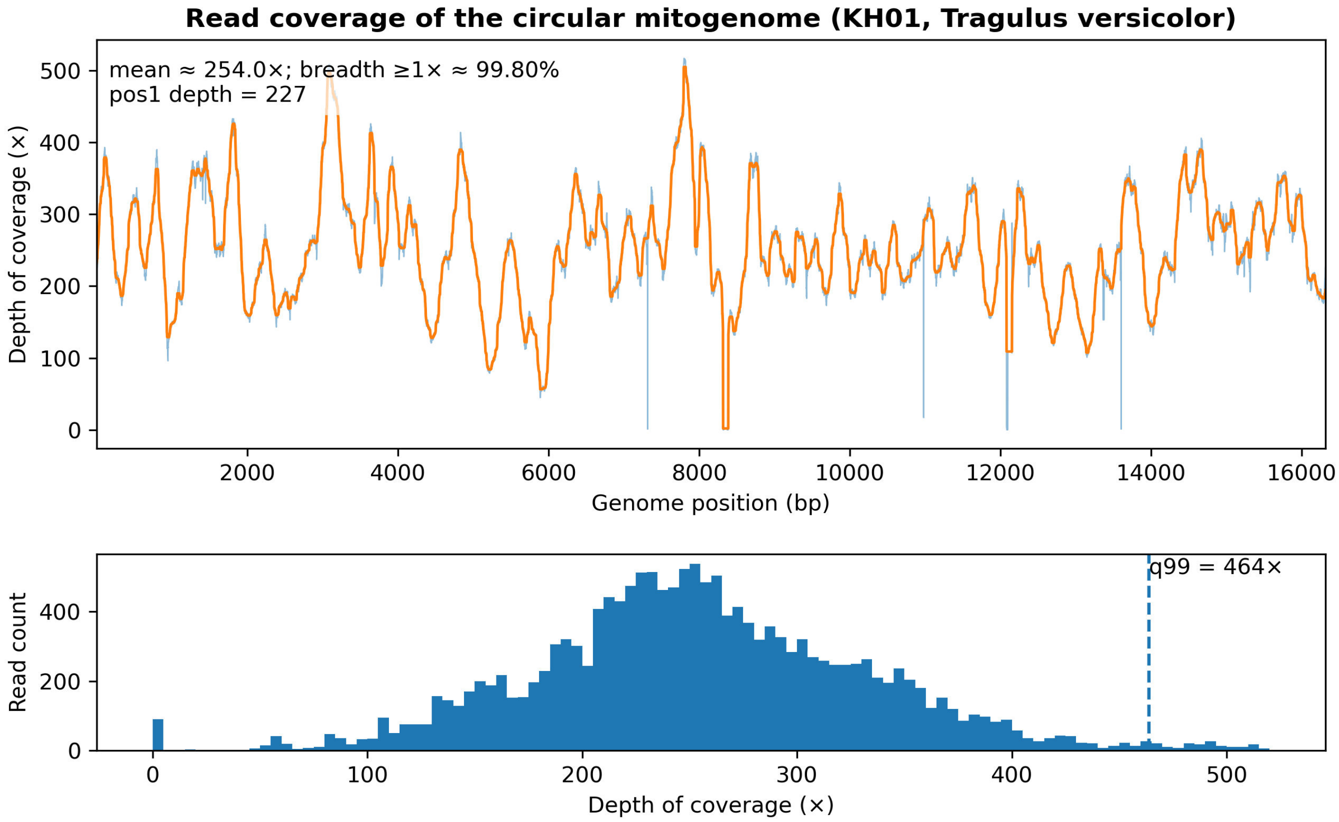

Supplementary Figure S1. Per-base read coverage of the circular mitochondrial genome of *Tragulus versicolor* (sample KH01, GenBank accession PV872897).

Clean paired reads were remapped to a circularized reference with the origin placed within the control region using BWA-MEM v0.7.17; BAM files were processed with samtools v1.19. Per-base depth was computed with samtools depth -a -J (counting positions overlapped by small deletions). To avoid edge artifacts, coverage was also verified on a twofold (2×) concatenated reference and folded back to the native genome length; positions are numbered from the circular origin. Top: per-base coverage (thin line) with a 50-bp rolling median (thick line). Bottom: depth histogram (q99 ≈ 464×). Summary: mean ≈ 254×; breadth (≥1×) ≈ 99.80%; depth at position 1 = 227×.
